# Characterization of the novel cross-genus phage vB_SmaS_QH3 and evaluation of its antibacterial efficacy against *Stenotrophomonas maltophilia*

**DOI:** 10.3389/fmicb.2025.1570665

**Published:** 2025-04-11

**Authors:** Peng Cheng, Zian Li, Lanmin Liu, Ruizhe Li, Jianwu Zhou, Xiaoqin Luo, Xiaoming Mu, Jingwei Sun, Jideng Ma, Xiangren A

**Affiliations:** ^1^Qinghai University, School of Clinical Medicine, Xining, China; ^2^Department of Clinical Laboratory, Qinghai Provincial People's Hospital, Xining, China

**Keywords:** *Stenotrophomonas maltophilia*, phage, genomic analysis, *Galleria mellonella*, phage therapy

## Abstract

**Background:**

Bacteriophages, which are natural bacterial predators, demonstrate potential as safe and effective biological control agents against drug-resistant infections. This study aims to characterize the biological properties of the novel lytic phage vB_SmaS_QH3 and comprehensively evaluate its efficacy in preventing and controlling clinically multidrug resistance *Stenotrophomonas maltophilia* infections using both *in vivo* and *in vitro* models.

**Methods:**

The phage was isolated from hospital sewage using the multidrug resistant *S. maltophilia* no. 3738 as the host. Transmission electron microscopy (TEM) was used to observe phage morphology, and the host range was determined via spot assays. Proliferation kinetics, including multiplicity of infection (MOI), adsorption rate, and one-step growth curves, were analyzed. Stability was assessed under various physicochemical conditions. Based on Illumina whole-genome sequencing data, bioinformatics tools were employed for gene annotation, functional prediction, and phylogenetic analysis. Antimicrobial activity was assessed using *in vitro* and *in vivo* models.

**Results:**

A lytic phage vB_SmaS_QH3 was isolated from hospital sewage. TEM revealed that it belongs to the class Caudoviricetes, featuring an icosahedral head (62 ± 3 nm) and a non-contractile long tail (121 ± 5 nm). Although the phage has a narrow host range, it exhibits cross-genus infectivity, lysing *S. maltophilia* (11/81) and *Pseudomonas aeruginosa* (3/24). The optimal MOI for phage vB_SmaS_QH3 is 0.01, with an adsorption rate of 49.16% within 20 min, a latent period of 40 min, a lytic period of 50 min, and a burst size of 41.67 plaque-forming units/cell. The phage remained stable at 4–60°C, at pH 3–11, and in chloroform, but it was completely inactivated following 20-min exposure to UV irradiation. Genomic analysis showed a linear double-stranded DNA genome of 43,085 bp with a GC content of 54.2%, containing 54 predicted ORFs, and no virulence or antibiotic resistance genes were detected. *In vitro*, vB_SmaS_QH3 effectively inhibited bacterial growth within 9 h. *In vivo*, it significantly improved the survival rate of *Galleria mellonella* larvae infected with *S. maltophilia*, regardless of the treatment timing.

**Conclusion:**

vB_SmaS_QH3 is a narrow host range lytic phage with a safe genome and excellent stability. It exhibits significant antibacterial activity both *in vitro* and *in vivo*, making it a promising candidate for therapeutic applications.

## 1 Introduction

*Stenotrophomonas maltophilia* is an emerging multidrug resistant opportunistic pathogen that is ubiquitous in nature and hospital environments (Majumdar et al., 2022). With the widespread use of broad-spectrum antimicrobial and immunosuppressive agents, as well as the increasing number of invasive operations in hospitals, the isolation rate of *S. maltophilia* has risen in recent years. It has become the third most common non-fermenting bacterium after *Pseudomonas aeruginosa* and *Acinetobacter baumannii* (Chang et al., 2015; Singhal et al., 2017). Despite its relatively low pathogenicity, *S. maltophilia*-related infections are most commonly observed in immunocompromised and critically ill patients, with pneumonia being the most common clinical presentation (Brooke, 2012, 2021; Hamdi et al., 2020; Gibb and Wong, 2021). Additionally, this strain can cause a variety of infections, including bloodstream, skin’s soft tissue, intra-abdominal cavity, urinary tract, and catheter- or implanted medical device-associated infections (Brooke, 2021). Furthermore, *S. maltophilia* causes community-acquired infections in healthy populations (Brooke, 2012; Mojica et al., 2022).

*S. maltophilia* infections are characterized by high mortality. Retrospective single-center studies have estimated all-cause mortality of 18–69% and attributable mortality of 24–58% at different time points after infection (Insuwanno et al., 2020; Mojica et al., 2022). Due to the natural multidrug resistance of the organism, clinical treatment options are limited, with sulfamethoxazole-trimethoprim (SXT) and levofloxacin (LEV) being the few effective treatment choices, of which SXT is preferred (Nicodemo and Paez, 2007). However, with the widespread use of antibiotics, *S. maltophilia* quickly develops antibiotic resistance by acquiring resistance genes (Liaw et al., 2010; Hu et al., 2011; Brooke, 2021). Hence, SXT- and LEV-resistant *S. maltophilia* is becoming a global epidemic. Kamuzu Central Hospital isolated multiple-drug resistance *S. maltophilia* in 2017 (Wang et al., 2020; Kumwenda et al., 2021). These pressing realities expose the formidable challenge of containing multidrug resistant *S. maltophilia* in clinical settings, which now demands immediate development of evolutionarily informed antimicrobial countermeasures to address its expanding resistance profiles.

Bacteriophages (phages), which are viruses that specifically infect bacterial hosts, are gaining prominence as promising antimicrobial agents (Jassim and Limoges, 2014). Their capacity to selectively target pathogenic bacteria while preserving the host microbiota, combined with minimal adverse effects and cost-efficient development, positions them as a compelling therapeutic alternative (McCutcheon and Dennis, 2021; Strathdee et al., 2023). However, certain biological characteristics of phages, such as host range, optimal number of infection replicates, temperature, and pH stability, limit their clinical applications and industrial production (Cai et al., 2019; Jończyk-Matysiak et al., 2019; Suh et al., 2022). Therefore, isolation and characterization of phages is essential for clinical application. Additionally, the safety and efficacy of phage therapies have been the focus of attention for clinicians and researchers. Moreover, preclinical evaluations through *in vitro* antimicrobial experiments and infected animal models are needed to simulate potential efficacy and provide guidance for clinical application (Liu et al., 2021; Gómez-Ochoa et al., 2022).

This study aimed to analyze complete genomic data and biological characterizations of phage vB_SmaS_QH3. Additionally, it sought to evaluate the *in vivo* prophylactic and therapeutic effectiveness of vB_SmaS_QH3 utilizing a *Galleria mellonella* (*G. mellonella*) larval infection model, serving as a valuable reference for future clinical applications.

## 2 Materials and methods

### 2.1 Bacteria strains and growth conditions

*S. maltophilia* and *P. aeruginosa* clinical isolates used in this experiment were collected from patient samples at Qinghai Provincial People’s Hospital. Bacterial isolation was performed using China Blue agar (Thermo Fisher Scientific, USA), followed by aerobic incubation at 37°C for 24 h, and species identification was achieved using matrix-assisted laser desorption/ionization time-of-flight mass spectrometry (MALDI-TOF MS EXS2600, Zybio, Chongqing, China, with a confidence threshold of ≥2.0). The host bacterium *S. maltophilia* no. 3738 for phage screening was isolated from sputum samples of a patient with chronic obstructive pulmonary disease and confirmed by Next-generation sequencing (BioProject: PRJNA1240898).

Antimicrobial susceptibility testing, conducted according to the CLSI M100 guidelines (2023 Edition) (Humphries et al., 2021), revealed that the strain exhibited a multidrug resistance sulfamethoxazole-trimethoprim (SXT, MIC = 80 mg/L) and levofloxacin (LEV, MIC ≥ 8 mg/L). Bacterial suspensions were prepared using n Luria-Bertani (LB) liquid medium (Haibo Biotechnology, Qingdao, China) and incubated in a constant-temperature shaker at 37°C (180 rpm) until reaching the logarithmic growth phase (OD600 = 0.6–0.8) for subsequent use.

### 2.2 Phage enrichment, isolation, and purification

The phage vB_SmaS_QH3 was successfully isolated from a sample of untreated wastewater sourced at the Qinghai Provincial People’s Hospital, employing *S. maltophilia* no. 3738 strain as the host. Phage enrichment, isolation, and purification were conducted using established methodologies and incorporating necessary adjustments for our specific study (Wen et al., 2022; Fang et al., 2023). Fresh untreated sewage samples from the hospital were collected by centrifugation at 10,000 × *g* for 5 min and filtered using a 0.22-μm filter (Millipore, USA) to obtain viral suspension. For phage enrichment, 10 mL of filtered supernatant was inoculated with 200 μL of logarithmic growth stage host cells into 10 mL of 2 × LB broth and incubated overnight at 37°C with 180 r/min shaking. The mixed culture was centrifugated at 10,000 × *g* for 5 min, followed by filtering the supernatant using a 0.22-μm filter to obtain a virus enrichment solution. Then, 100 μL of the virus-enriched solution and 100 μL of the logarithmic growth phase *S. maltophilia* no. 3738 bacterial suspension were mixed into 5 mL of 1.5% LB agar and poured onto an LB agar plate. The resulting plaques were observed after overnight incubation at 37°C. A clear phage plaque was selected on the host bacterial lawn and diluted with phosphate buffered saline (PBS) buffer to the appropriate ratio to obtain viral dilution. Afterward, 100 μL of the viral dilution and 100 μL of the logarithmic growth phase *S. maltophilia* no. 3738 bacterial suspension were mixed into 5 mL of 1.5% LB agar and spread onto an LB agar plate. The size and shape of the plaques were observed after overnight incubation in a 37°C incubator. The above-described isolation steps were repeated until obtaining phage plaques of uniform size and morphology. The purified phages were stored in the LB broth containing 50% glycerol at −80°C for subsequent experiments.

### 2.3 Matrix-assisted laser desorption/ionization-time of flight mass spectrometry (MALDI-TOF MS) and host range

Fresh single colonies were selected and spread directly onto the specimen wells of the MALDI-TOF MS target plate. Each target spot was covered with a drop of 1-μL 70% formic acid. After drying at room temperature, 1 μL of matrix solution was added to each target spot. Then, again after drying at room temperature, the target plates were loaded onto a MALDI-TOF MS EXS2600 for mass spectrometry data acquisition. After the acquisition, the mapping data of the target strains were imported into EX-Smartspec software (Zybio, Chongqing, China) for cluster analysis. Subsequently, cluster tree diagrams were drawn to visualize the similarities and differences between the strains. Finally, in conjunction with the host profiles of vB_SmaS_QH3, we used the iTOL v6^1^ online tool for graphical beautification and presentation to facilitate a clearer presentation of the results.

The host range of vB_SmaS_QH3 was determined using a spot test (McCutcheon et al., 2020) against 81 clinical strains of *S. maltophilia* and 24 strains of *P. aeruginosa*, as well as three clinical isolates of *Burkholderia cepacia* (*B. cepacia*). Briefly, 5 μL of filtered phage suspension (10^10^ plaque-forming unit [PFU]/mL) was poured onto petri dishes covered with different bacterial lawns. The plates were incubated overnight at 37°C, and the plaques were observed and categorized the next day with the following standard (Kutter, 2009): 4+ for completely cleared; 3+ for clear but with a faint hazy background; 2+ for clear but with a faint hazy background; 1+ for a few isolated plaques or a severe hazy background; and 0 for not cleared.

### 2.4 Transmission electron microscopy

Phages were amplified using the double-layer agar method. Once numerous plaques formed, LB broth was added to submerge the agar layer. The mixture was shaken at 100 rpm for 4 h to elute phage particles. The eluate was transferred to a centrifuge tube and centrifuged at 5,000 × *g* for 10 min to remove bacterial debris. The supernatant was collected, filtered through a 0.22-μm filter, and the purified lysate was obtained. Phage titers were determined using the double-layer agar method, and only lysates with titers >10^9^ PFU/mL were selected as the preservation solution for subsequent experiments. Twenty μL of the phage preservation solution was dropped onto a copper grid to dry naturally for 10 min, and the excess liquid was blotted out with filter paper. Subsequently, 20 μL of the 2% phosphotungstic acid solution was dropped onto the copper grid to negatively stain for 5 min, and the staining solution was blotted out with filter paper and dried at room temperature for 30 min. Then, it was photographed using a transmission electron microscope (TEM, Hitachi HT7700, Japan) at 80 kV. Head width and tail length of virus particles were measured using Image-Pro Plus 6.0.

### 2.5 Optimal multiplicity of infection

Multiplicity of infection (MOI) refers to the ratio of phages to host bacteria during the course of infection (Ji et al., 2019). In the experiment, the initial concentration of *S. maltophilia* no. 3738 was set at 10^8^ CFU/mL. One mL of phage vB_SmaS_QH3 suspension (with concentrations ranging from 10^10^ to 10^4^ PFU/mL) was mixed with an equal volume of bacterial suspension, resulting in final MOIs of 100, 10, 1, 0.1, 0.01, 0.001, and 0.0001. The mixtures were then incubated overnight at 37°C with shaking at 180 rpm. Phage titers were determined using the double-layer plate method, and the MOI with the highest phage titer was the optimal MOI (Gong et al., 2016). The experiment was conducted three times independently.

### 2.6 Adsorption rate

Adsorption rate was measured following literature methods (Han et al., 2021; Wang et al., 2024) and then optimized according to preliminary experimental results. The steps were as follows: 10 mL of *S. maltophilia* no. 3738 (10^8^ CFU/mL) was mixed with 10 mL of phage vB_SmaS_QH3 (10^6^ PFU/mL) to achieve a MOI of 0.01, then incubated at 37°C with shaking at 200 rpm. During this period, 1 mL of mixed culture was withdrawn at 0, 2, 4, 6, 8, 10, 15, 20, 25, and 30 min, respectively, using a syringe, and filtered through a 0.22-μm filter. Phages bound to bacteria were retained, while free phages passed into the filtrate. The titer of free phages in the filtrate was determined by the double-layer agar plate method. Adsorption rate = [(Initial phage titer − Phage titer in the filtrate)/Initial phage titer] × 100%. We repeated the experiment three times for reliability.

### 2.7 One-step growth curve

One-step growth experiments were performed according to a previous study with some modifications. One mL of logarithmic growth phase *S. maltophilia* no. 3738 suspension and 1 mL of vB_SmaS_QH3 solution were mixed at the optimal MOI ratio. Adsorption was achieved by shaking at 37°C and 180 rpm for 20 min, followed by centrifugation of the mixture at 4°C and 10,000 × *g* for 10 min. The precipitate was washed and resuspended with PBS, centrifuged again at 4°C and 10,000 × *g* for 10 min. Then, the above-mentioned step was repeated once again. The bacterial pellet was resuspended in 10 mL of LB liquid medium and incubated at 37°C with continuous shaking at 180 rpm. Samples were taken every 10 min up to 120 min to monitor phage titer via the double-layer plate method. The experiment was performed in triplicate, and phage titers from three independent replicates were averaged.

### 2.8 Physicochemical stability

#### 2.8.1 Temperature stability

Temperature stability testing of the vB_SmaS_QH3 phage was performed following a previously described methodology with appropriate adaptations (Han et al., 2021). The phage was diluted to 10^8^ PFU/mL with saline magnesium (SM) buffer (pH 7.5). Then, 1 mL of the phage diluent was placed in a sterile centrifuge tube and water-bathed for 1 h at 4, 25, 37, 50, 60, 70, and 80°C. Finally, the phage titer was determined using the double-layer plate method. This experiment was conducted in triplicate.

#### 2.8.2 pH stability

In separate 900-μL centrifuge tubes, we evaluated phage stability under different pH conditions using SM buffers with pH ranging from 1 to 14 (Bai et al., 2022). First, the phage suspension was diluted with the LB broth to 10^9^ PFU/mL. Subsequently, 100 μL of the phage suspension was added to each tube containing 900 μL of different pH buffers, and then the tubes were incubated in a water bath at 37°C for 1 h. This experiment was conducted in triplicate.

#### 2.8.3 UV radiation stability

Phage stability under ultraviolet (UV) radiation was tested according to a previous report (Ahmad et al., 2024) with slight modifications. Briefly, the phage suspension was diluted to 10^8^ PFU/mL in the SM buffer. Then, 90-mm sterile, uncovered petri dishes were placed in a biosafety cabinet, 30 cm away from the UV light source (light intensity: 196.67 ± 28.27 μW/cm^2^). Subsequently, 10 mL of the phage dilution was added to the UV radiation–exposed petri dish. Samples of phage dilutions were collected at 10 min intervals for 1 h to determine their potency using a double agar covered plaque test method. This experiment was conducted in triplicate.

#### 2.8.4 Chloroform stability

We tested the sensitivity of the phage to chloroform to determine whether the viral capsid contains lipids (Ahmad et al., 2024). Chloroform was mixed with a phage suspension (10^8^ PFU/mL) to reach final concentrations of 0, 1, 2, 4, and 5%. After 1 h in a 37°C water bath, phage titer was performed using the LB double-layer plate method. This experiment was conducted in triplicate.

### 2.9 Phage genome sequencing and bioinformatic analysis

Phage DNA was extracted using the QIAamp DNA Mini Kit (Qiagen, Hilden, Germany) according to the manufacturer’s protocol. The whole genome was sequenced using Illumina’s MiSeq sequencing platform (Thermo Fisher Scientific, United States) and the PromethION sequencer (Oxford Nanopore Technologies, Oxford, UK) (Kozich et al., 2013). Quality control and low-quality sequences were filtered by fastp (Version: 0.23.2) (Chen et al., 2018). Then, the sequence was assembled using Unicycler (Version: 0.5.0) (Wick et al., 2016). Coding gene prediction of the assembled genome was performed by Prokka (Version: 1.14.6) (Seemann, 2014). The online tool RAST^2^ (Aziz et al., 2008) was used to predict and annotate open reading frames (ORFs). tRNA gene prediction was conducted using the tRNA scan-SE program (Chan and Lowe, 2019). Antimicrobial resistance genes (AMRs) and virulence genes were screened in the Comprehensive Antibiotic Resistance Database^3^ (Alcock et al., 2022) and the VirulenceFinder^4^ (Liu et al., 2018). Sequence similarity analyses and comparisons were performed using the NCBI BLAST algorithm. The lifestyle of the phage was predicted using the Phage AI platform, version 1.0.2 (Tynecki et al., 2020). The phylogenetic analysis of phage vB_SmaS_QH3 was performed utilizing VICTOR (Meier-Kolthoff and Göker, 2017).^5^ Concurrently, the average nucleotide identity (ANI) was determined through VIRIDIC (Moraru et al., 2020).^6^

### 2.10 Bacterial cell killing assay *in vitro*

Assessment of the lysis effect of phage vB_SmaS_QH3 on host bacteria using *in vitro* inhibition assays (Li et al., 2020). Phages were added to host bacterial cultures (OD 600 = 0.2) at MOIs of 100, 10, 1, 0.1, and 0.01, followed by incubation at 37°C on a shaker at 180 rpm. Bacterial cultures without phages were used as untreated group controls. The OD 600 values of the cultures were measured using a NanoPhotometer N50 (Implen, Germany) every 1 h for 12 h. The experiment was performed independently in triplicate.

### 2.11 Evaluation of phage antimicrobial efficacy *in vivo*

This study chose *G. mellonella* larvae (Huiyude Biotech Company, Tianjin, China) as an animal model to assess phage antibacterial effects *in vivo*. Healthy 5th-instar larvae from the same batch, with a body length of 25 ± 5 mm and weight of 300 ± 25 mg, were selected to establish the animal model.

Establish a *G. mellonella larval* bacterial infection model to assess the virulence of *S. maltophilia* no. 3738 and determine its appropriate lethal dose in larvae (Li et al., 2023). A total of 50 larvae were selected for this experiment. The surface was sterilized with 75% ethanol swabs. The experiment was divided into two major groups: PBS negative control group and bacterial infection group. The PBS negative control group contained 10 larvae and was only injected with PBS. The bacterial infection group was divided into four subgroups, with 10 larvae in each subgroup, which were injected with log-phase *S. maltophilia* no. 3738 at concentrations of 8 × 10^5^ CFU/mL, 4 × 10^6^ CFU/mL, 2 × 10^7^ CFU/mL, and 1 × 10^8^ CFU/mL, respectively. A 10-μL bacterial solution or PBS was injected into the last left proleg using a Hamilton microsyringe (Hamilton, Shanghai, China) to select an appropriate infection dose. After injection, move the larvaes into a sterile petri dish that has a sterile filter paper, and subsequently hatched in a constant temperature incubator at 37°C with 60 ± 10% relative humidity (RH) in the dark. When *G. mellonella* larvae did not respond to touch, they could be considered dead (Wei et al., 2016). Survival rates were observed and recorded every 8 h over a total observation period of 72 h. This experiment was repeated in triplicate.

The *G. mellonella* larval infection model was used to assess the therapeutic and preventive effects of phage vB_SmaS_QH3 on *S. maltophilia* infections (Li et al., 2023). In the experiment, logarithmic-phase *S. maltophilia* no. 3738 was washed with PBS and diluted to 1 × 10^8^ CFU/mL to prepare the bacterial suspension. In the therapeutic experiment, 70 larvae were selected, surface-sterilized with 75% ethanol swabs, and randomly divided into seven groups: negative control group (10 larvae), bacterial infection control group (10 larvae), and phage treatment group (50 larvae). The negative control group was injected with 10 μL of PBS first, and then injected with 10 μL of MOI 100 (10^10^ PFU/mL) phage after 1 h to evaluate the toxicity of the phage itself; the bacterial infection control group was injected with 10 μL of bacterial suspension first, and then injected with 10 μL of PBS after 1 h; the phage treatment group was divided into five subgroups, corresponding to MOIs of 100, 10, 1, 0.1, and 0.01, respectively. Each larva was first injected with 10 μL of bacterial suspension, and then injected with phage suspension at different concentrations (1 × 10^10^, 1 × 10^9^, 1 × 10^8^, 1 × 10^7^, and 1 × 10^6^ CFU/mL) after 1 h. The preventive experiment was similar to the therapeutic experiment, with the same injection doses, but the injection order was reversed: the negative control group was injected with phage first, then PBS; the bacterial infection control group was injected with PBS first, then bacterial suspension; the preventive group was divided into five subgroups, which were injected with phage at different concentrations (same MOIs as the treatment group), and then injected with bacterial suspension after 1 h. After each injection, the larvae were transferred to petri dishes with sterile filter paper and incubated in the dark. Survival rates were recorded every 8 h for a total observation period of 72 h. All experiments were performed in triplicate. The larval mortality data from the experiments were entered into GraphPad Prism software for plotting survival curves and statistical analysis.

### 2.12 Statistical analysis

**Figure 1:**
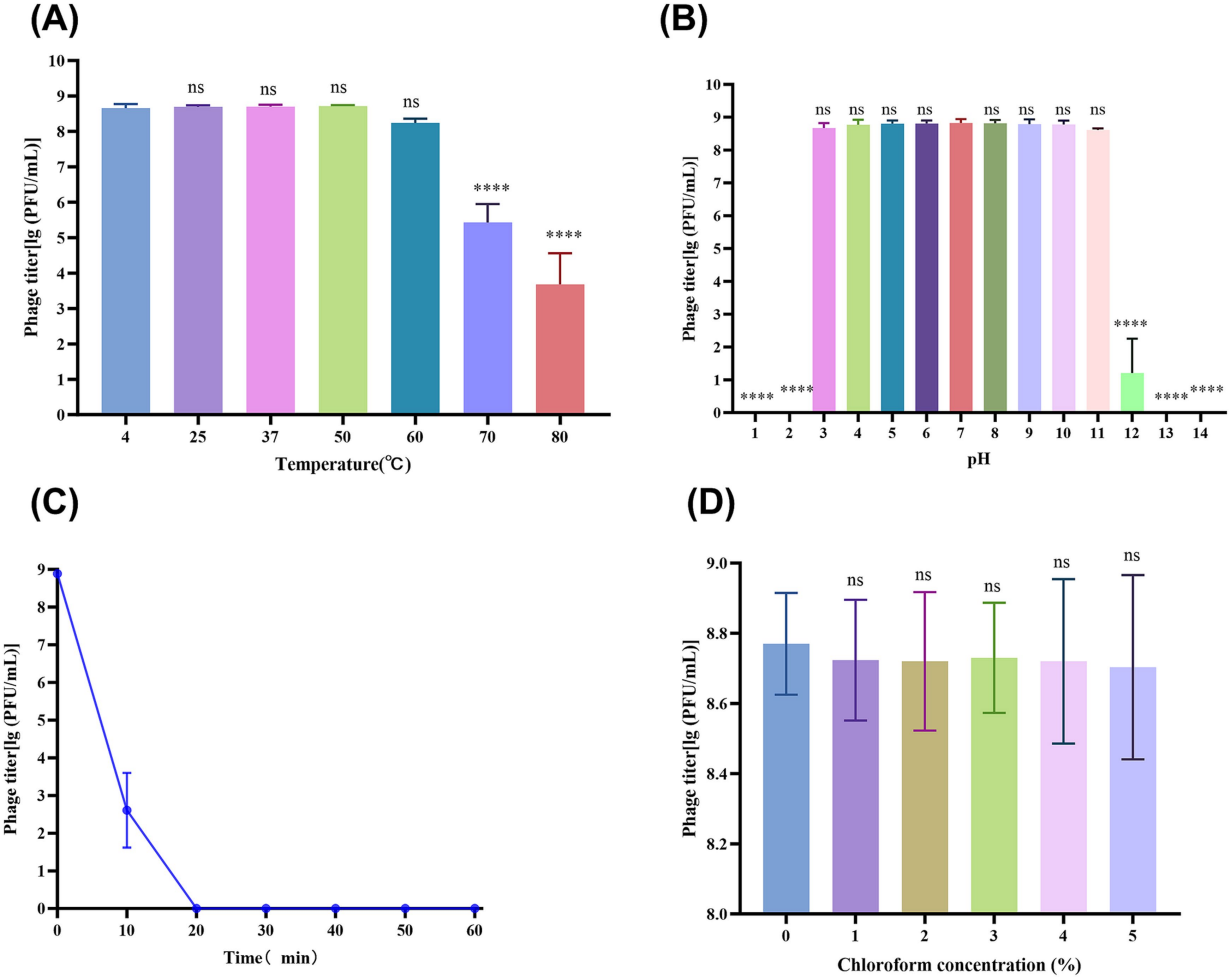
vB_SmaS_QH3 physiochemical influences. **(A)** vB_SmaS_QH3 temperature stability. **(B)** vB_SmaS_QH3 stability at different pH values. **(C)** vB_SmaS_QH3 stability against UV radiation. **(D)** Effect of various chloroform percentages on vB_SmaS_QH3. Figures 1A,B,D: Statistical analysis performed using one-way ANOVA. Significance levels indicated as: ns for non-significance; *p < 0.05; **p < 0.01; ***p < 0.001; ****p < 0.0001.

**Figure 2:**
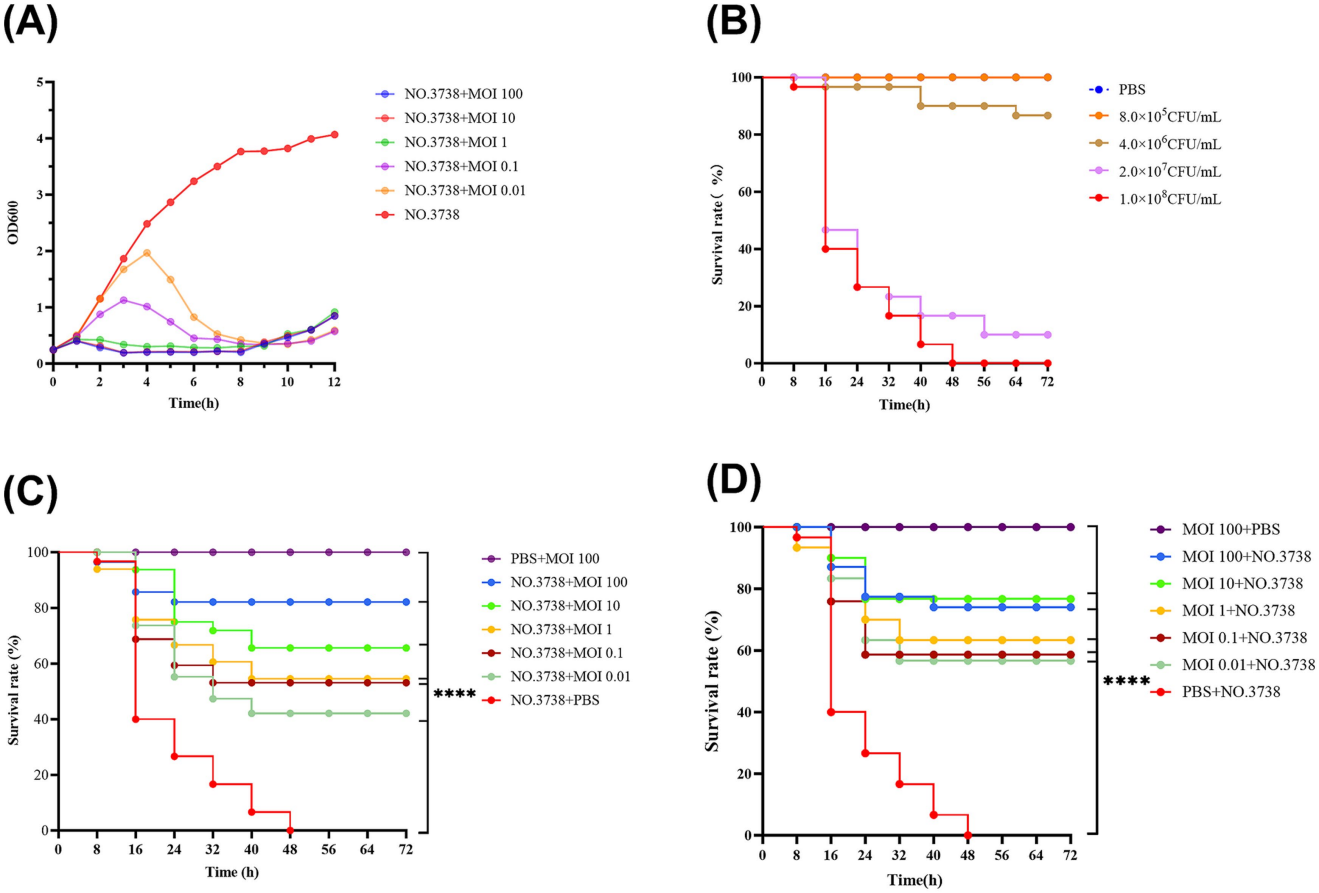
Antimicrobial effects of the phage *in vitro* and *in vivo*. **(A)** Lytic activity of vB_SmaS_QH3 against *S. maltophilia* no. 3738 at different MOIs; **(B)** Survival curve of *G. mellonella* after infecting with different *S. maltophilia* no. 3738 concentrations; **(C)** Survival rate during phage treatment; **(D)** Survival rate after phage prophylaxis. ****indicates statistical significance at *p* < 0.0001 (Mantel-Cox).

All data were analyzed using GraphPad Prism 10.1.2 software and are expressed as a mean and standard deviation. One-way ANOVA was employed for Figures 1A,B,D, while the survival curves of *G. mellonella* in Figures 2C,D were analyzed using the log-rank (Mantel-Cox) test. A *p*-value <0.05 was considered statistically significant.

## 3 Results

### 3.1 Isolation and morphological characteristics

**Figure 3:**
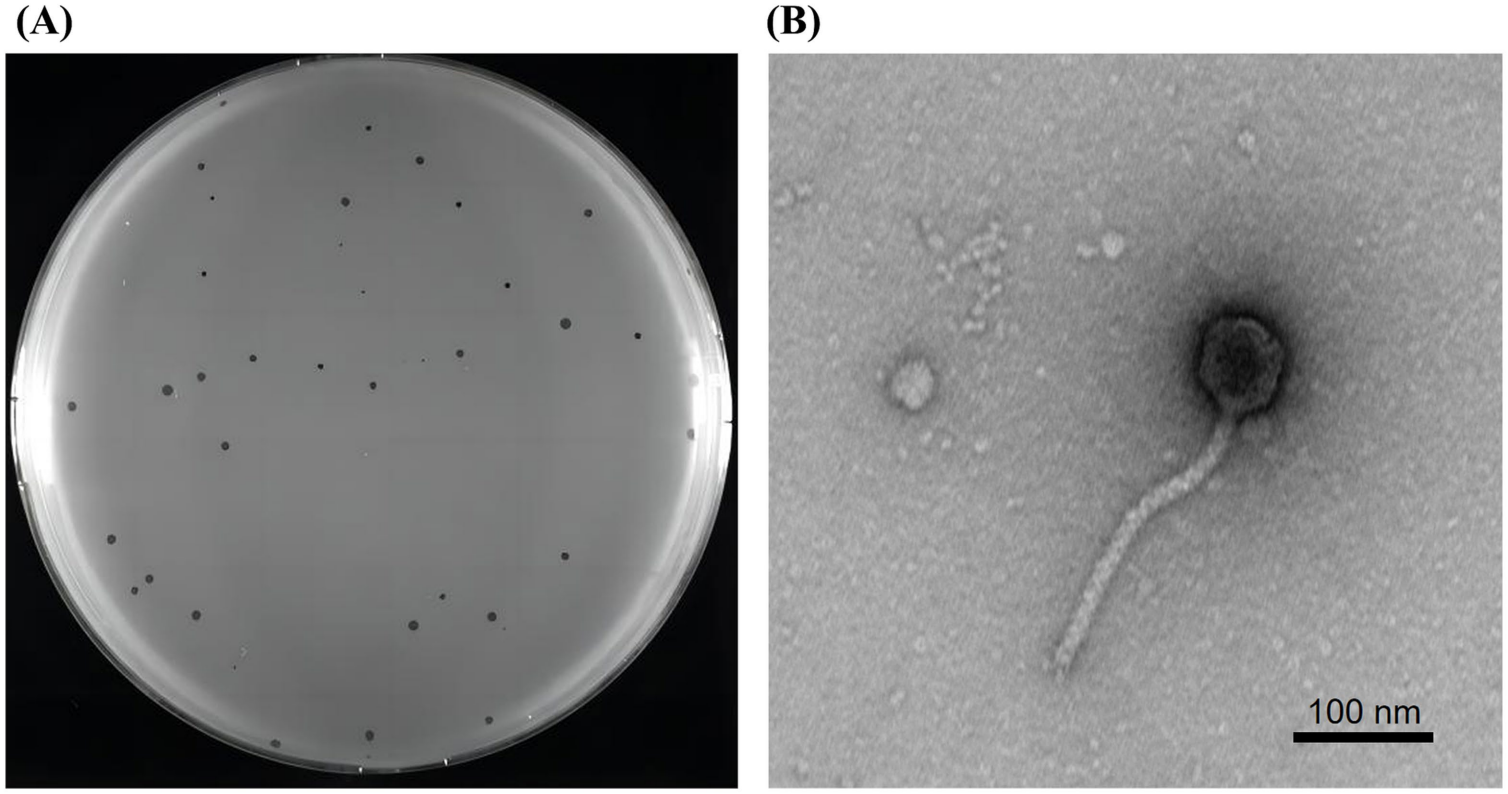
Plaques and transmission electron microscopy of phage vB_SmaS_QH3. **(A)** Plaque morphology of phage vB_SmaS_QH3 on a bacterial lawn of *S. maltophilia* no. 3738 in a double-layer agar. **(B)** Transmission electron microscopy of phage vB_SmaS_QH3. Scale bar, 100 nm.

A lytic phage, vB_SmaS_QH3, hosted by *S. maltophilia* no. 3738, was successfully isolated from untreated hospital wastewater, forming inhomogeneous transparent plaques of 0.5–2 mm in diameter on double-layer plate without a “halo” phenomenon (Figure 3A). The characterization of this inhomogeneous patch remained consistent throughout multiple purifications and subsequent experiments. TEM showed that phage vB_SmaS_QH3 had a symmetric icosahedral head (50.86 ± 1.10 nm) and a non-contractile long tail (166.97 ± 6.65 nm) (Figure 3B). According to morphology and the latest criteria of the International Committee on Classification of Viruses (ICTV) in the 2024 release, vB_SmaS_QH3 belongs to the class Caudoviricetes.

### 3.2 MALDI-TOF MS and host range

**Figure 4:**
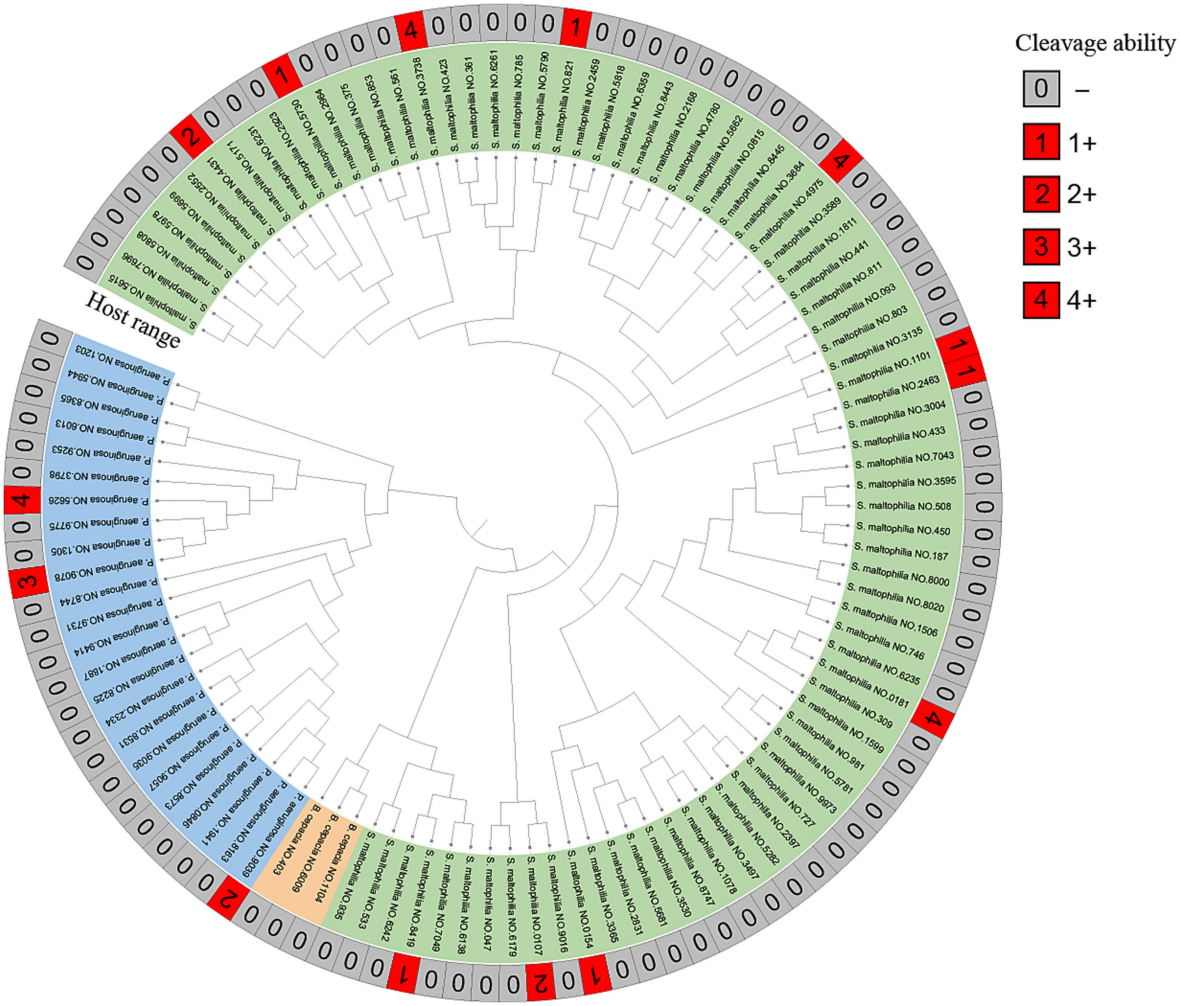
MALDI-TOF MS of clinical strains and host range of vB_SmaS_QH3. Developmental trees were established by cluster analysis using MALDI-TOF fingerprinting. The cleavage spectra were judged as follows: 4+ for completely cleared; 3+ for the entire area clear but with faint haze in the background; 2+ for the entire area clear but with faint haze in the background; 1+ for a few isolated plaques or a severe hazy background; 0 for not cleared (−).

Phage vB_SmaS_QH3 showed a narrow host range among the tested strains of clinically derived *S. maltophilia*, infecting only 11 of 81 strains. The phage had the unique infection ability across the bacterial taxonomic genus, being able to infect 3 of 24 *P. aeruginosa* strains (Figure 4). Partial plaque assay results of the phage’s host range are shown in Supplementary Figure 1. However, it did not show infectivity against any of the 3 *B. cepacia* strains.

### 3.3 MOI, adsorption, and one-step growth curve analysis

**Figure 5:**
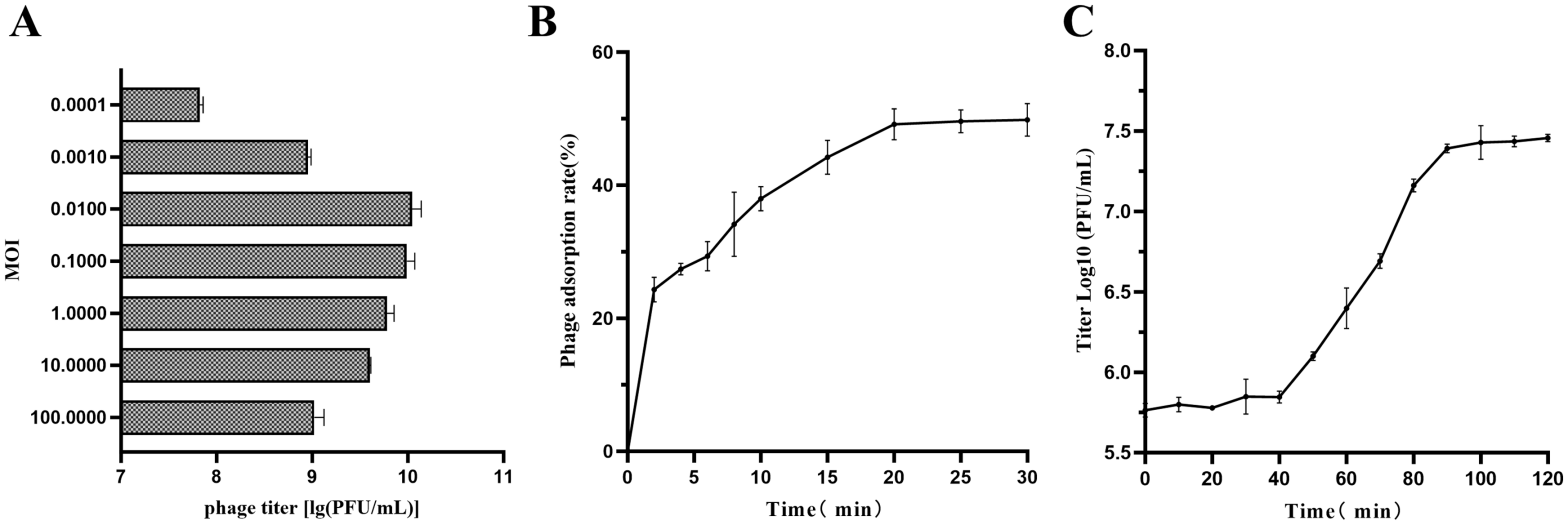
Growth characteristics of vB_SmaS_QH3. **(A)** The phage titers under different MOIs (phage/bacteria = 0.0001, 0.001, 0.01, 0.1, 1, 10, 100) are depicted on the x-axis. **(B)** Adsorption rate of vB_SmaS_QH3 (MOI = 0.01). **(C)** One-step growth curve test of vB_SmaS_QH3 was performed at MOI = 0.01.

The optimal MOI experiment showed that the peak titer of vB_SmaS_QH3 was 1.12 × 10^10^ PFU/mL when MOI = 0.01, indicating that the optimal MOI of vB_SmaS_QH3 was 0.01 (Figure 5A). As shown by the adsorption curve of vB_SmaS_QH3 to *S. maltophilia* no. 3738, the phage progressively adsorbs to host cells, achieving an adsorption rate of 49.16% by 20 min post-infection, after which the rate stabilizes (Figure 5B). Determination of the phage one-step growth curve revealed that the phage vB_SmaS_QH3 latency period was about 40 min, with the lysis phase ending at the 90th min to enter the plateau phase. The burst size was 41.67 PFU/cell (Figure 5C).

### 3.4 Physicochemical stability

Thermal stability tests showed that the vB_SmaS_QH3 titer remained stable at ambient temperatures ranging from 4°C to 60°C, indicating that vB_SmaS_QH3 has good temperature stability. After treatment at 70°C and 80°C for 1 h, the phage titer significantly decreased (*p* < 0.0001) by 3 and 5 orders of magnitude, respectively (Figure 1A). The phage was incubated for 1 h in the SM buffer at different pH values ranging from 1 to 14 to assess the phage stability at different pH values. There was no significant change in the titer of phage under a pH of 3–11. However, when the pH increased to 12, the phage titer decreased sharply (*p* < 0.0001). At pH of 1, 2, 13, and 14, the phages were completely inactivated (Figure 1B). vB_SmaS_QH3 is highly sensitive to UV light: after 1 h of UV irradiation, the phage titer was drastically reduced to less than 0.01% and became completely undetectable after 20 min (Figure 1C). vB_SmaS_QH3 exposure to different concentrations of chloroform for 1 h resulted in a slight decrease in phage potency. Specifically, the logarithmic values of phage titers decreased by 9.22, 8.68, 9.40, 7.41, and 9.04% at chloroform concentrations of 1, 2, 3, 4, and 5%, respectively (Figure 1D). However, there was no statistically significant difference compared with the control group that did not receive chloroform. The results indicated that vB_SmaS_QH3 was less sensitive to chloroform, which confirms that this phage might be the lipid-free phage (Selcuk et al., 2024).

### 3.5 Genomic analysis and functional annotation

**Figure 6:**
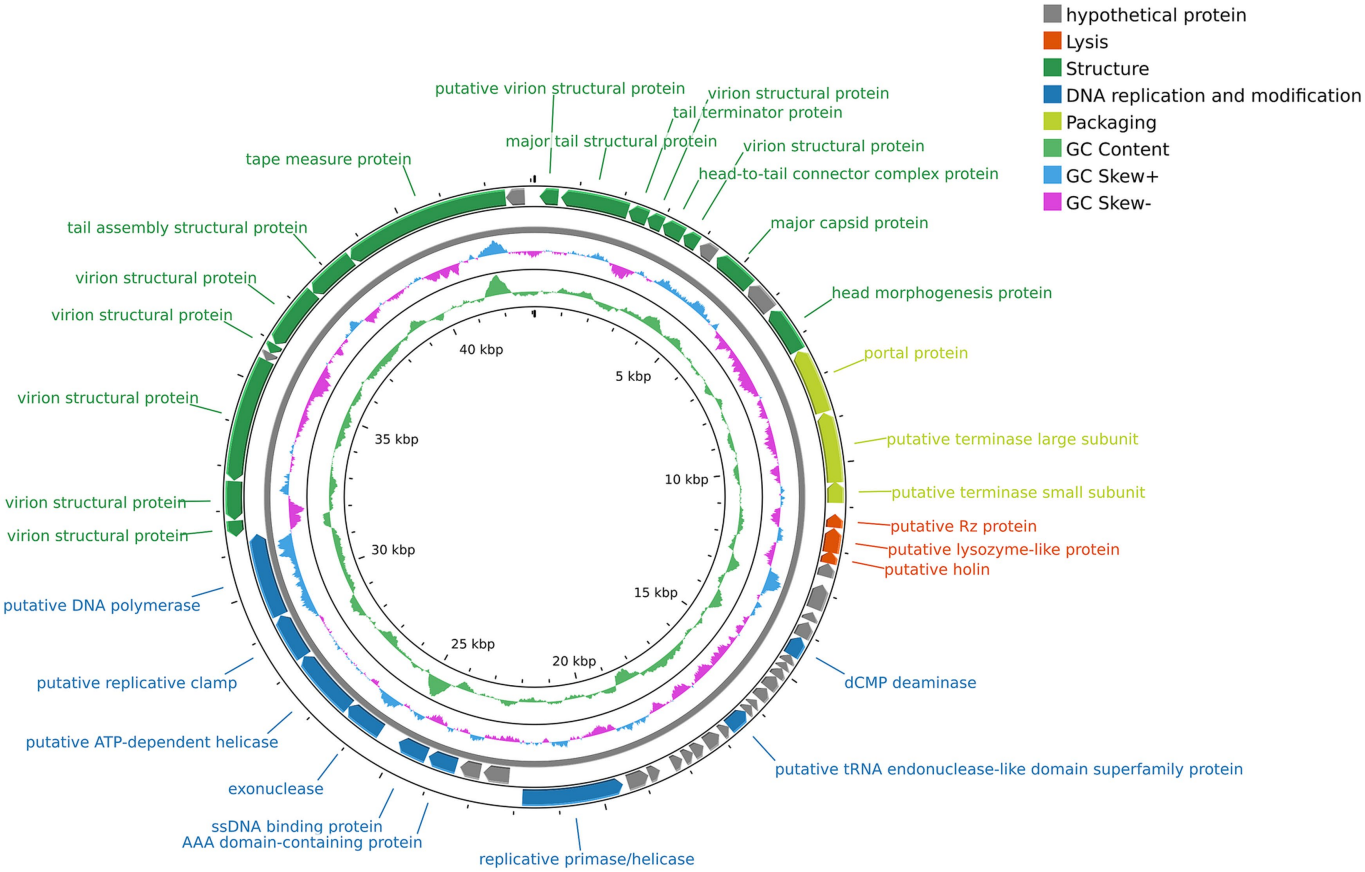
Genome map of phage vB_SmaS_QH3. The outermost circle represents the ORFs. Different colors represent genes with different functions: gray, hypothetical protein; red, lysis; deep green, structure; deep blue, DNA replication and modification; light green, packaging.

The complete genome sequence of vB_SmaS_QH3 was submitted to GenBank with the accession number PP932004. Genome sequencing showed that vB_SmaS_QH3 is a linear double-stranded DNA with a 43,085-bp size and a 54.2% GC content. RAST annotation showed that the genome contains 54 ORFs, with 8 ORFs located on the positive strand and 46 ORFs located on the negative strand (Supplementary Tables 1–3 and Figure 6). All ORFs had ATG as the start codon. The total length of the ORFs was 40,179 bp (average length: approximately 744 bp), with a high gene density of 93.26%. The genome of vB_SmaS_QH3 lacks tRNA, virulence genes, antibiotic resistance genes, and genes related to lysogeny formation. Furthermore, the prediction of a “Virulent” lifestyle by Phage AI collectively indicate that vB_SmaS_QH3 is a lytic phage.

The genome annotation of phage vB_SmaS_QH3 revealed 54 open reading frames (ORFs), among which 30 were assigned predicted functions, while the remaining 24 were classified as hypothetical proteins (Figure 6 and Supplementary Table 3). Based on the functional predictions of the 30 protein-encoding ORFs, the genome of phage vB_SmaS_QH3 can be categorized into four distinct modules. The first module includes 15 ORFs associated with phage structure, such as putative virion structural proteins (ORF1, ORF4, ORF6, ORF46, ORF47, ORF48, ORF50, ORF51), major tail structural protein (ORF2), tail terminator protein (ORF3), head-to-tail connector complex protein (ORF5), major capsid protein (ORF8), head morphogenesis protein (ORF10), tail assembly structural protein (ORF52), and tape measure protein (ORF53). The second module comprises nine ORFs involved in DNA replication and modification, including putative tRNA endonuclease-like domain superfamily protein (ORF29), exonuclease (ORF42), dCMP deaminase (ORF21), AAA domain-containing protein (ORF40), ssDNA binding protein (ORF41), replicative primase/helicase (ORF37), putative ATP-dependent helicase (ORF43), putative replicative clamp (ORF44), and putative DNA polymerase (ORF45). The third module contains three ORFs related to packaging, such as putative portal protein (ORF11), putative terminase large subunit (ORF12), and putative terminase small subunit (ORF13). The fourth module includes three ORFs associated with lysis, namely putative Rz protein (ORF14), putative lysozyme-like protein (ORF15), and putative holin (ORF16). This functional classification provides a comprehensive understanding of the genomic organization and potential biological roles of phage vB_SmaS_QH3.

### 3.6 Phylogenetic analysis

**Figure 7:**
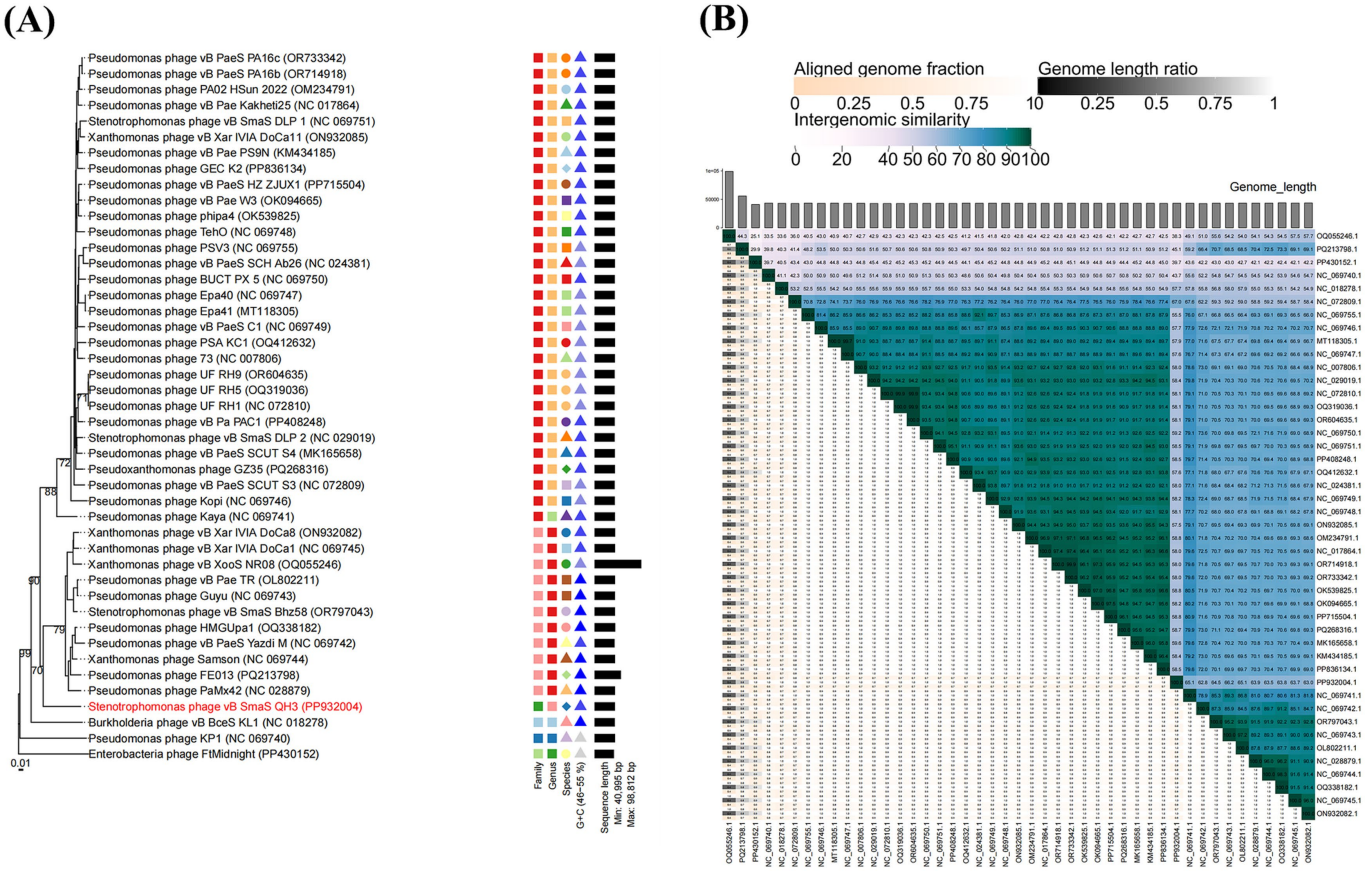
Phylogenetic analysis of phage vB_SmaS_QH3. **(A)** A phylogenetic tree was generated using the whole genome sequence through VICTOR analysis, suggesting that phage vB_SmaS_QH3 may represent a novel species. **(B)** Percentage sequence similarity between phages calculated using VIDIRIC shows that phage vB_SmaS_QH3 could be a novel clade. The horizontal and vertical axes indicate the corresponding phage GenBank number.

A total of 44 phages in the NCBI database were highly homologous to vB_SmaS_QH3 (E-value of 0%). We constructed a phylogenetic tree of the whole genome sequences of 45 phages including vB_SmaS_QH3 to investigate the evolutionary history of phage vB_SmaS_QH3 and its relationship with other phages. The GBDP tree extrapolated from formulas D0, D4, and D6 showed an average support of 1, 22, and 3%, respectively. Therefore, formula D4 with the highest support was selected for further analysis. The 45 phages were categorized into 40 species-level clusters, seven genus-level clusters, and six family-level clusters (Figure 7A). Phage vB_SmaS_QH3 showed the closest relationship with *Pseudomonas* phage PaMx42, a member of the class Caudoviricetes. Additionally, 44 phages homologous to phage vB_SmaS_QH3 belong to the same genus according to the VICTOR classification of families, genera, and species. According to NCBI records, these phages are all categorized in the class Caudoviricetes. However, at the species level, there were 45 distinct clusters in which vB_SmaS_QH3 independently represents a species (Figure 7A). Based on VIRIDIC’s whole-genome average nucleotide identity (ANI) analysis of phage vB_SmaS_QH3 and 44 others, vB_SmaS_QH3 shows only 66.2% similarity to Pseudomonas phage Guyu, far below the species (95%) and genus (70%) demarcation thresholds (Adriaenssens and Brister, 2017) (Figure 7B). Moreover, VIRIDIC assigns vB_SmaS_QH3 to species_cluster 27 and genus_cluster 7 (Supplementary Table 4), distinct from other phages. These data indicate significant genomic differences in vB_SmaS_QH3, supporting its classification as a new genus.

### 3.7 Antibacterial effect of vB_SmaS_QH3 *in vivo* and *in vitro*

The *in vitro* inhibitory effect of phages with different MOIs (100, 10, 1, 0.1, and 0.01) was assessed by measuring OD 600 over a 12-h period using bacterial cultures without phages as a control (Figure 2A). The OD 600 of the control group increased consistently over 12 h, while the addition of phage vB_SmaS_QH3 at different MOIs resulted in significant differences in OD 600 values over time. Infections of *S. maltophilia* no. 3738 with vB_SmaS_QH3 at various MOIs showed a slight increase in OD 600 values during the first 1 h. Subsequently, OD 600 values decreased and remained low after infection at MOI of 100, 10, and 1. However, at MOIs of 0.1 and 0.01, OD 600 values continued to increase until the 3rd and 4th h, respectively, before declining rapidly. By the 9th h, the OD 600 values of the infected groups under all MOI conditions started increasing, indicating that phage-resistant bacteria might have emerged and proliferated between the 8th and 9th h.

Survival graphs of *G. mellonella* showed that *G. mellonella* larvae injected with only PBS and a high phage concentration (10^10^ PFU/mL) did not show any death or body darkening (Figures 2B,C,D), suggesting that PBS and phage are safe for the larvae. In the experiment, we used different concentrations of bacterial suspensions to infect *G. mellonella* larvae. However, when the infective dose was increased to 1 × 10^8^ CFU/mL, the larvae exhibited rapid mortality within 24 h, with mortality progressively increasing to achieve 100% by 48 h (Figure 2B), which ensures the controllability of the experimental process. Therefore, we chose 1 × 10^8^ CFU/mL as the infectious dose for evaluating the therapeutic efficacy of phage vB_SmaS_QH3 against *S. maltophilia* infections (Figure 2B).

In the phage-treated infected *G. mellonella* larvae model, phage-treated larva survival was enhanced at all time points. Specifically, the 72-h survival of *G. mellonella* larvae infected with phage at MOIs of 100, 10, 1, 0.1, and 0.01 was 83.33, 63.33, 50.00, 40.00, and 26.67%, respectively. Thus, Phage could boost the survival of infected *G. mellonella* (*p* < 0.001) (Figure 2C). In the phage prophylaxis model, pre-injection of phage also enhanced the survival of infected *G. mellonella*, with 72-h survival of 73.33, 76.67, 63.33, 56.67, and 56.67%, respectively. Hence, pre-injecting phage vB_SmaS_QH3 was effective in preventing infection in the host (*p* < 0.001) (Figure 2D).

## 4 Discussion

*S. maltophilia*, a naturally multidrug resistant bacterium, is classified by the World Health Organization as one of the leading hospital-acquired drug-resistant pathogens in the world, posing a major threat to global public health (Brooke, 2013; Li et al., 2023). Therefore, developing new strategies to combat drug-resistant bacteria is urgently needed. Furthermore, phage therapy showed great potential in combating drug-resistant bacterial infections, demonstrating good safety and efficacy (Gordillo Altamirano and Barr, 2019; Hatfull et al., 2022; Strathdee et al., 2023). With the aggravation of the bacterial drug resistance problem, the research and development of new phage-based therapeutics are greatly important for the prevention and treatment of drug-resistant bacterial infections. Consequently, phage-related research is expected to be increasingly emphasized in the future. In this research, we effectively isolated a novel phage (designated vB_SmaS_QH3) targeting multidrug resistance *S. maltophilia*. This study comprehensively examined phage vB_SmaS_QH3’s biological properties and whole genome data, and assessed its *in vitro* and *in vivo* antibacterial effects.

On a double-layer agar, vB_SmaS_QH3 could form transparent plaques with a 0.5–2 mm diameter. TEM analysis confirmed that this phage is a member of the class Caudoviricetes. The whole genome of phage QH3 showed <95% homology with any phage in the NCBI phage database by the BLASTn comparison analysis. According to the ICTV criteria (Adriaenssens and Brister, 2017), suggesting that it may be a novel phage. Additionally, using BLAST comparison with the NCBI database, the whole genome of phage vB_SmaS_QH3 showed generally high similarity to *P. aeruginosa* phage, in addition to its high similarity to a *Burkholderia* phage KL1 (accession: NC_018278.1) strain (similarity: 82.25%, query cover: 62% with E-value: 0). Therefore, to explore the host range of phage vB_SmaS_QH3, we not only tested 81 *S. maltophilia* strains but also added 24 *P. aeruginosa* and 3 *B. cepacia* strains. Our research results show that phage vB_SmaS_QH3 has marked host specificity for *S. maltophilia*, lysing only 11 of the 81 strains tested. In contrast, it could lyse some *P. aeruginosa strains* (3/24). However, the phage did not infect *B. cepacia*. We are aware that from 1961 to 1983, *S. maltophilia* was classified as a member of the genus Pseudomonas (Said et al., 2025). This classification indicates a close phylogenetic relationship between *S. maltophilia* and *P. aeruginosa*, suggesting that they may share certain surface protein receptors. In the future, we plan to identify the common receptors on these two bacteria that are recognized by the phage vB_SmaS_QH3. This research will be of great significance for elucidating the mechanisms of phage cross-species properties and expanding the host range. Phages are generally considered to have relatively narrow species specificity, and those capable of crossing species boundaries for infection are extremely rare, including phages DLP1 (accession: KR537872) and DLP2 (accession: KR537871) (Peters et al., 2015), phage PhiOT8 (Evans et al., 2009), and phage Kpp95 (Wu et al., 2007). Therefore, we speculate that the similarity of phage genomes may be related to their host range. vB_SmaS_QH3 demonstrates cross-genus infectivity, capable of infecting bacteria from various taxonomic genera. This makes it particularly valuable for treating mixed bacterial infections or infections caused by unknown bacteria. However, we must be vigilant about the similarity of phage genomes to prevent phages from crossing species boundaries and adversely affecting the normal microbiota when exploring phage therapy. Furthermore, bacteria are more likely to develop resistance to phages with a narrow host spectrum because these phages rely on a limited number of specific host molecules or structures for infection. As a result, bacteria can acquire resistance through a single or a few mutations. In contrast, broad-spectrum phages target a wide range of hosts, requiring bacteria to undergo multiple mutations or develop complex mechanisms to achieve resistance, which significantly increases the difficulty of resistance development (Labrie et al., 2010). Therefore, Hence, when applying vB_SmaS_QH3 in different fields, it is crucial to carefully consider the limitations of its narrow host range and phage resistance.

Multilocus sequence typing (MLST) is a gold standard for bacterial typing, yet its high cost and time-consuming nature restrict its extensive use (Larsen et al., 2012). By contrast, MALDI-TOF MS, known for its simplicity, speed, and cost-effectiveness, has become the top choice for clinical microbial identification and an alternative to MLST for bacterial typing (Sauget et al., 2017). Given the link between MLST and phage host bacteria, and the connection between MALDI-TOF MS and MLST in bacterial typing, it’s logical to explore MALDI-TOF MS’s potential in phage host-range prediction (Yamamura et al., 2022). However, initial studies fail to show a significant link between host bacterial protein fingerprinting and phage-host relationships, highlighting the complexity of phage-host interactions and the inadequacy of protein fingerprints alone. To dig deeper, we’ll select phages and hosts with specific MLST characteristics, merge MLST and MALDI-TOF MS data, and use machine learning to build an association model. This can enhance our understanding of phage-host interactions and may offer a fast and accurate way to screen host bacteria for phage therapy.

The pH and temperature of phage storage are critical factors affecting their activity and stability (Malik et al., 2017). vB_SmaS_QH3 demonstrated remarkable stability across a broad pH range (3–11) and temperature (4–60°C). This observation aligns with earlier studies indicating that members of the Siphoviridae family possess significant resilience in adverse conditions (Lasobras et al., 1997). Excellent temperature and pH stability of the phage, which is important for its production, transportation, storage, and application, also highlights its suitability and potential in diverse applications (Malik et al., 2017). Additionally, we found that phage vB_SmaS_QH3 is extremely sensitive to UV light and can be almost completely inactivated by only 10 min of UV irradiation, with a sensitivity similar to that of vB_XooS_NR08, which is also almost completely inactivated after 5-min UV irradiation (Jain et al., 2023). Genomic analysis revealed that vB_SmaS_QH3 shares high genetic homology with vB_XooS_NR08 (query cover: 72%, E-value: 0, identities: 85.63%). This high genetic similarity confirms their close association at the molecular level and may explain their similar sensitivity to UV light. Furthermore, morphological comparisons under TEM showed a high structural similarity between the two phages (Jain et al., 2023). Thus, vB_SmaS_QH3 should be protected from sunlight and UV light during storage and use given the significant effect of UV light on phage activity. vB_SmaS_QH3 has a significant tolerance to chloroform, suggesting that it may be a lipid-free phage (Li et al., 2024).

The MOI of a phage is a key indicator for measuring its lysis efficiency. A lower optimal MOI indicates that fewer phages are required to lyse the same number of bacteria (Bai et al., 2022). In this study, the optimal MOI of the phage vB_SmaS_QH3 was 0.01, which is similar to the optimal MOI of the *S. maltophilia* phage BUCT609 reported in the literature (Han et al., 2022). This result suggests that at an MOI of 0.01, each phage can produce the maximum number of progeny, thereby achieving the highest proliferation efficiency. In industrial-scale phage production, the optimal MOI is typically used for phage cultivation to achieve high-titer production targets. This approach not only reduces production costs but also enhances economic efficiency (João et al., 2021). From the adsorption curve of phage vB_SmaS_QH3, it can be seen that its adsorption process is relatively slow. At 20 min, the adsorption rate is only close to 50%, and then it stabilizes. In contrast, many phages exhibit faster adsorption speeds, such as phage BUCT555 reaching 90% adsorption within 10 min (Han et al., 2021), and phage BUCT700 achieving close to 90% adsorption in 16 min (Li et al., 2023). Therefore, the adsorption performance of vB_SmaS_QH3 is not ideal. Studies have shown (Marcó et al., 2010; Tomat et al., 2022) that various physicochemical factors significantly affect the adsorption efficiency of phages. To address this, we plan to optimize experimental conditions in the future to improve its adsorption efficiency. This will help to more comprehensively understand the applicable scope of vB_SmaS_QH3 and is of significant importance for evaluating its effectiveness in different application scenarios.

The latent period of vB_SmaS_QH3 is approximately 40 min, whereas the lytic period is about 50 min. Additionally, phage lysis time is closely related to holin (Catalão et al., 2011), a class of small hole-forming transmembrane proteins encoded by double-stranded DNA phages. After phage infection, holin mediates the lysis of bacteria and is known as the molecular timer of bacterial lysis (Reddy and Saier, 2013). Holin (ORF16) forms transmembrane pores in host cell membranes, initiating osmotic and solubilization processes (Palmer et al., 2020). ORF15 encodes a lysozyme-like protein with a function similar to that of lysozymes, which specifically degrades peptidoglycan in the bacterial cell wall. Furthermore, the holin-formed pore facilitates the entry of lysozyme-like proteins into the bacterial interior, promoting the rupture of the host cell (Kim et al., 2020). The synergistic action of these two proteins precisely modulates the timing of lysis, ensuring that phage particles are released at the optimal time while avoiding premature destruction of the host cell, which could affect phage replication. ORF14 encodes the Rz protein, a transmembrane protein of phage origin with peptidase activity (Summer et al., 2007). It can damage the cell wall and membrane of the host bacterium, promoting the rapid release of progeny phages. Through the intensive study of phage lysis-related enzymes, these enzymes are expected to become potential alternatives to broad-spectrum antibiotics (Song et al., 2020).

In addition, the vB_SmaS_QH3 genome contains 15 structurally related genes, mainly distributed at both ends of the genome sequence. Major capsid protein (ORF8) and head morphogenesis protein (ORF10) are involved in the formation of phage head (Rao and Black, 2010; Ignatiou et al., 2019; Davis et al., 2022). Furthermore, ORF2, ORF3, ORF52, and ORF53 encode the major tail structural protein, the tail terminator protein, the tail assembly structural protein, and the tape measure protein, respectively, all of which are involved in the formation of the phage tail. The genes facilitating phage head formation are typically highly conserved, ensuring a seamless phage life cycle. In contrast, the genes encoding the phage tail are more prone to mutations, allowing them to adapt to the varying recognition requirements of different bacteria, thereby altering their structure (Dion et al., 2020). The head-to-tail connector complex protein encoded by ORF5 connects the capsid to the tail (Das et al., 2018).

Furthermore, the DNA packaging motor of vB_SmaS_QH3 comprises three protein components: portal protein (ORF11), small subunit (ORF13), and large subunit (ORF12). ORF12, ORF13, and ORF11 all showed the highest homology to *Pseudomonas* phage PaMx42 (JQ067092) of 90.9, 82.7, and 91.2%, respectively. Portal proteins are usually located at the apex of the five-fold symmetry axis in the head of the phage and are the key proteins connecting the head and the tail, acting as the entry and exit points for the DNA channel (Prevelige and Cortines, 2018). Terminase is an essential protein involved in DNA packaging, typically including large subunit (ORF12) and small subunits (ORF13) that are adjacent to each other. Both subunits play crucial roles in the splicing and packaging of phage DNA. The large subunit is primarily responsible for ATP-driven DNA translocation, while the small subunit interacts with the large subunit of terminase, specifically binding and cleaving near the initial site to initiate packaging (Feiss and Rao, 2011; Hawkins et al., 2024).

Finally, replication is a complex process involving multiple proteins. The nine ORFs of phage vB_SmaS_QH3 encode proteins that are directly involved in or assist phage replication to ensure its accuracy and efficiency, including dCMP deaminase (ORF21), putative tRNA endonuclease-like domain superfamily protein (ORG29), replicative primase/helicase (ORF37), replicative primase/helicase (ORF37), AAA domain-containing protein (ORF40), ssDNA binding protein (ORF41), exonuclease (ORF42), putative ATP-dependent helicase (ORF43), putative replicative clamp (ORF44), and putative DNA polymerase (ORF45). In the phage replication cycle, ORF37 and ORF43 encode helicase-related proteins that use the energy of ATP hydrolysis to unwind dsDNA and form a single-stranded template during replication initiation (Guilliam et al., 2015). The DNA polymerase encoded by ORF45 is a key enzyme in phage DNA replication, being responsible for synthesizing new DNA strands and repairing damaged DNA (Morcinek-Orłowska et al., 2022). Phage exonuclease is involved in DNA repair, being able to recognize and excise damaged or faulty nucleotides (Fu et al., 2024).

In summary, the genomic analysis of phage vB_SmaS_QH3 revealed the lack of virulence-associated genes, antibiotic resistance-related genes, and lysogeny-associated genes. Furthermore, Phage AI predicted phage vB_SmaS_QH3 as having a “Virulent” lifestyle. Combined with its phenotypic characteristics, we confirm that phage vB_SmaS_QH3 is a lytic phage. These findings demonstrate its genetic safety and highlight its enhanced potential for practical applications.

Pairwise sequence similarity and phylogenetic analysis are key criteria for defining virus taxonomic groups (Lefkowitz et al., 2017). NCBI blastn of phage vB_SmaS_QH3’s whole-genome sequence against Pseudomonas phage PaMx42 (JQ067092) shows the highest similarity, with 85.63% nucleotide identity and 72% coverage, indicating a novel genome. VICTOR’s phylogenetic analysis confirms the close relationship between vB_SmaS_QH3 and PaMx42, yet vB_SmaS_QH3 forms a distinct branch. To determine if vB_SmaS_QH3 constitutes a new genus, we used VIDIRIC for ANI analysis with 44 other phages. Results show vB_SmaS_QH3 shares only 66.2% similarity with Pseudomonas phage Guyu (NC_069743), below the species (95%) and genus (70%) thresholds. Thus, vB_SmaS_QH3 is confirmed as a new genus.

*In vitro* experiments showed that the inhibitory effect of the phage on bacteria changes dynamically over time. At the beginning of the experiment (1–9 h), phages exhibited significant inhibitory activity, likely due to the intense “arms race” between phages and host bacteria (Hampton et al., 2020). Phages gradually gained dominance through rapid replication and release, effectively inhibiting bacterial growth. However, the emergence of phage-resistant strains over time suggests the development of resistance mechanisms in the host bacteria. In the control group that was not infected with phage vB_SmaS_QH3, bacterial growth was not inhibited. In contrast, phage-treated groups with different MOIs (0.01, 0.1, 1, 10, and 100) demonstrated significant bacterial inhibition, with the effect becoming more pronounced as the MOI increased. Although phages with different MOIs induced the emergence of resistant mutant strains, the proliferation of these mutant strains did not differ according to MOI. This phenomenon was also verified in other *S. maltophilia* phages, such as A1432 (Li et al., 2024) and BUCT700 (Li et al., 2023). The emergence of phage resistance suggests the need to consider the development of resistance in host bacteria and its impact on therapeutic efficacy in phage therapy.

*G. mellonella* larvae represent an ideal model for studying immune responses due to their low cost, ease of handling, innate immune responses similar to those of vertebrates, and the absence of ethical issues (Insua et al., 2013; Wei et al., 2016). This study investigated the antimicrobial effect of phage vB_SmaS_QH3 *in vivo* using a *G. mellonella* larvae infection model. Phage vB_SmaS_QH3 was non-toxic to larvae, and a single injection of phage showed better efficacy for prophylaxis and treatment, with prophylaxis providing a more prominent effect. vB_SmaS_QH3 anti-infective safety and efficacy results were consistent with the results of previous studies (Marongiu et al., 2022; Pu et al., 2022), indicating that phage is a safe and effective anti-infective agent.

## 5 Conclusion

Phage vB_SmaS_QH3, a newly discovered virulent phage belonging to the class Caudoviricetes, exhibits cross-genus infectivity despite its narrow host range, being capable of lysing both *S. maltophilia* and *P. aeruginosa*. Genomic analysis, biological characterization, and *in vitro* and *in vivo* experimental results demonstrate that vB_SmaS_QH3 possesses excellent stability and lytic activity. Furthermore, its genome lacks virulence, resistance, and lysogenicity-related genes, ensuring its safety for potential applications. Although its narrow host range may limit its broad-spectrum utility, vB_SmaS_QH3 holds significant value as a potential candidate for the prevention and treatment of *S. maltophilia* infections. Future research could further explore its application potential by optimizing its host range.

## Data Availability

The original contributions presented in the study are publicly available. This data can be found at: https://www.ncbi.nlm.nih.gov/nuccore/PP932004. The names of the repository/repositories and accession number(s) can be found in the article/Supplementary material.

## Footnotes

^1^ https://itol.embl.de/

^2^ https://rast.nmpdr.org/

^3^ https://card.mcmaster.ca/analyze

^4^ http://www.mgc.ac.cn/VFs/

^5^ https://ggdc.dsmz.de/victor.php#

^6^ https://rhea.icbm.uni-oldenburg.de/VIRIDIC/
