# Supplementary material for: Characterization of the novel cross-genus phage vB_SmaS_QH3 and evaluation of its antibacterial efficacy against Stenotrophomonas maltophilia
Source: Front Microbiol. 2025 Apr 11;16:1570665. doi: 10.3389/fmicb.2025.1570665 (PMC12023781; doi:10.3389/fmicb.2025.1570665)
Supplement: Supplementary file 1 [file Data_Sheet_1.docx]

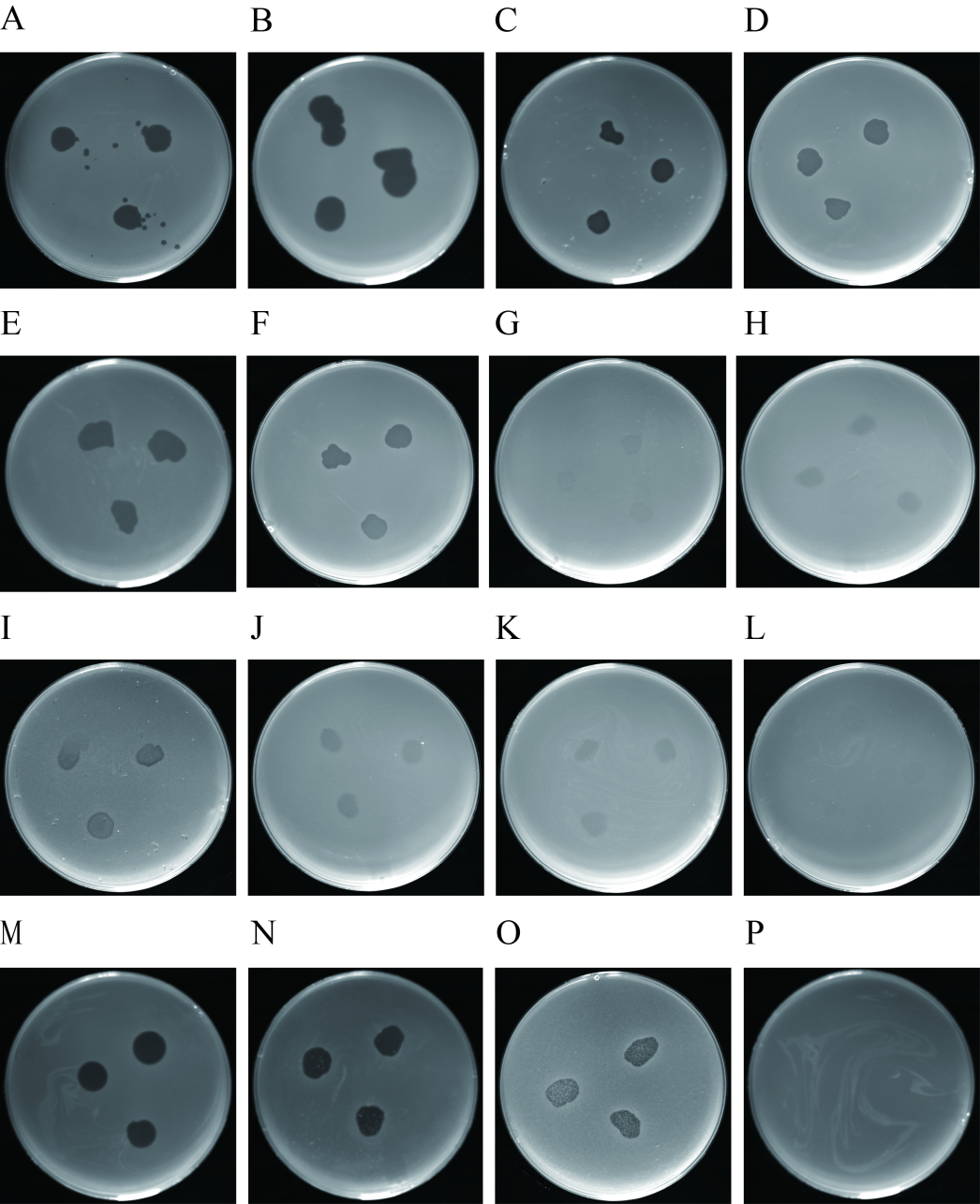
Supplementary Material

**Supplementary Figure 1.** Plaque Assay Showing the Host Range of Phage vB_SmaS_QH3.(A): *S. maltophilia* no. 3738 (4+):. (B): *S. maltophilia* no. 0181 (4+):(C): *S. maltophilia* no. 4975 (4+). (D): *S. maltophilia* no. 0107 (2+). (E): *S. maltophilia* no. 4431 (2+). (F): *S. maltophilia* no. 5730 (1+). (G): *S. maltophilia* no. 0154 (1+). (H): *S. maltophilia* no. 821 (1+). (I): *S. maltophilia* no. 1101 (1+). (J): *S. maltophilia* no. 2463 (1+). (K): *S. maltophilia* no. 8419 (1+). (L): *S. maltophilia* no. 727 (0). (M): *P. aeruginosa* no. 5626 (4+). (N): *P. aeruginosa* no. 9078 (3+). (O): *P. aeruginosa* no.9039 (2+). (P): *P. aeruginosa* no. 8531 (0). and the plaques were observed and categorized the next day with the following standard:4+ for completely cleared; 3+ for clear throughout but with a faint hazy background; 2+ for clear throughout but with a faint hazy background; l+ for a few isolated plaques or severe turbidity; and 0 for not cleared severe hazy background.
